# Reliability and validity of the Turkish version of the digital competence questionnaire for nurses

**DOI:** 10.1186/s12912-025-03052-6

**Published:** 2025-09-30

**Authors:** Arzu Bulut, Reyhan İnce Kasap, Nuray Yılmaz

**Affiliations:** 1 Faculty of Health Sciences, Department of Health Management, Bandirma Onyedi Eylul University, Bandirma, Balikesir, 10200 Türkiye; 2 Director of Health Care Services, Republic of Turkiye Ministry of Health, Bulancak State Hospital, Giresun, Türkiye; 3 Director of Health Care Services, Republic of Turkiye Ministry of Health, Giresun Training and Research Hospital, Giresun, Türkiye

**Keywords:** Digital competence, Digital health skills, Knowledge, Attitude, Skills, Nursing digital proficiency, Nursing education, Psychometric validation

## Abstract

**Background:**

Digital competence is crucial for nurses adapting to technological advancements and ensuring effective patient care. The purpose of this study was to evaluate the reliability and validity of the Turkish version of the Digital Competence Questionnaire (DCQ), thus providing a scientific basis for its study application among Turkish nurses.

**Methods:**

A cross-sectional validation study was conducted with 423 registered nurses from two hospitals in the Black Sea region Turkiye using convenience sampling. Two independent samples were collected: 208 participants for exploratory factor analysis (EFA) and 215 participants for confirmatory factor analysis (CFA). The data were collected through online surveys, assessing demographic characteristics, digital competence, attitudes towards digital technology, and affinity for technology interaction. The Turkish version of DCQ was created by translating and revising the original scale, conducting item and factor analysis, and validating it with validation factor analysis.

**Results:**

The Turkish version of the DCQ demonstrated a two-factor structure: Knowledge and Skills, and Attitude. EFA and CFA confirmed the scale’s construct validity. The CFA model fit indices indicated an excellent fit with the data. Reliability measures were high, with internal consistency and test-retest reliability. Scores on the Turkish version of the DCQ were significantly associated with technology interaction and attitudes toward digital technology. The Turkish DCQ showed robust psychometric properties, aligning with the theoretical framework of digital competence.

**Conclusions:**

The Turkish adaptation of the DCQ is a valid and reliable instrument for assessing nurses’ digital competence. It provides a valuable resource for identifying gaps in digital skills and designing targeted interventions in clinical practice. The tool also supports efforts to enhance digital literacy and integration of digital technologies within nursing education and practice in Turkiye.

**Clinical trial number:**

Not applicable.

**Supplementary Information:**

The online version contains supplementary material available at 10.1186/s12912-025-03052-6.

## Introduction

Today, beyond its enormous impact on our daily lives, digitalization significantly impacts how we work due to the application of new technologies to working life [1, 2]. Professionally used devices and software programs are constantly being upgraded to make work easier and more efficient. These changes require the increased use of digital information and communication technology (ICT) in the workplace. The healthcare sector is among the sectors most affected by this development as a knowledge intensive sector. Advancing digitalization in healthcare is increasingly affecting the daily work of nurses in different roles. Health information technologies and applications designed to support nurses in these roles include electronic staffing, workload measurements, nursing processes, decision-making, and nursing documentation [3].

Despite these advances, there are still many gaps between what is desired and what is available and used in the various settings where nurses work [4]. Large amounts of patient information documented by nurses can be used in secondary analyses to improve patient outcomes and the quality and efficiency of care processes. However, these data are rarely used. The reasons for this may be the poor quality of nursing documentation due to a lack of knowledge and skills in the nursing process or technical and organizational barriers in nursing information systems or computer-assisted care documentation [5, 6]. Another example of these gaps is electronic information dissemination and access via social media [7]. All these higher-level deficit areas are often associated with more essential skills and the motivation to develop and use them. Sometimes, this can be part of a negative feedback loop. For example, if nurses are unfamiliar with analyzing electronic and structured data for quality assurance, they may not be interested in developing documentation skills. This argument can also work the other way; nurses who do not have appropriate skills in electronic documentation with nursing terminologies, may not recognize opportunities to transfer patient information to different media and may not consider using patient data for research and quality assurance. In another example, a lack of in-depth knowledge of data protection and an unjustified fear of violating rules can be barriers to adopting health IT systems. Although there have been great achievements and outstanding practices, most nurses and other health professionals have not sufficiently accepted and internalized these technologies and their impact. Based on all these arguments, it is possible to say that nurses need enhanced digital competence to use technologies appropriately in their workplace.

Digital competence involves a variety of skills and competences, and its scope is wide, covering media and communication, technology and computing, literacy, and information science [8]. It refers to confident, critical, and creative use of information and communication technologies (ICT) to accomplish objectives associated with employability, learning, leisure activities, social inclusion, and social participation. More broadly, digital competence encompasses the body of knowledge, skills, attitudes, and competencies required to use ICT and digital media effectively, efficiently, appropriately, critically, creatively, ethically, flexibly, autonomously, and intentionally to perform tasks, solve problems, communicate, manage information, collaborate, create and share content and build knowledge. This competence aims to enable individuals to act effectively in different contexts such as work, learning, socializing, consumption and empowerment [9].

In the healthcare sector, recognizing the impact of digital competence is crucial, as a lack of competence among healthcare professionals can compromise patient safety, increase the frequency of errors [10], and negatively affect service quality [11]. Moreover, it may reduce the willingness to use and implement new digital tools [12], which could lead to stress and burnout among healthcare workers [11].

The factors influencing healthcare professionals’ digital health competencies are multifaceted. Colleagues, the work environment, managers, orientation processes, and continuous training opportunities can either support or hinder the development of digital health competence [13, 14]. Additionally, healthcare professionals’ motivation, willingness to adopt digital tools and their previous experience of using them have been found to influence not only their digital health competence but also for further development of their competence [13, 15]. Greenhalgh et al. [16] emphasize that for the successful adoption of digital health technologies, continuous training and internal support systems for employees are crucial. Furthermore, the development of digital competencies requires healthcare professionals to adapt to this transformation, highlighting the importance of education and support programs [16]. Achieving adequate competence in digitalization necessitates regular assessment, training, and education of healthcare professionals, all of which fall under the responsibility of healthcare institutions [10].

More evidence is needed in the literature about nurses’ knowledge, skills, or behaviors related to their digital competencies in clinical practice. In Turkiye, the increasing digitalization of healthcare highlights the critical need for nurses to develop and demonstrate digital competence. Turkiye has one of the largest patient record systems in the world and continues to invest in technological solutions in public healthcare. It is therefore essential that nurses are confident and knowledgeable in using digital tools and making sure that they use the system competently. However, the literature on this subject remains limited, particularly regarding reliable and validated tools for assessing the digital competencies of nurses. Existing studies have primarily focused on other professional groups, such as educators [17–22], leaving a significant gap in understanding the unique digital skills required in nursing. This gap is especially concerning, given the pivotal role of nurses in patient care and the growing reliance on digital systems in clinical practice.

The significance of this research lies in its potential to inform educational and policy initiatives aimed at enhancing digital knowledge, skills, and attitudes in nursing. As healthcare continues to evolve, ensuring that nurses possess the necessary digital competencies is critical for maintaining high standards of patient care and fostering innovation in the field. Digital Competence is a relatively novel concept that researchers have studied [1, 8, 9, 11]. Although digital competence questionnaires exist, a few instruments are specifically designed to measure knowledge, skills, and attitudes together. For instance, the reliability and exploratory factor structure were tested, but the validity of the Digital Competence Questionnaire was not validated among nurses [23, 24]. Despite the critical role of digital competence in nursing, there is a lack of validated tools to assess nurses’ digital skills, particularly in the Turkish context. Therefore, a new questionnaire is needed to measure the digital competence of Turkish nurses in clinical practice, addressing knowledge, skills, and attitudes. Therefore, the current study aims to bridge this critical gap by evaluating the psychometric properties of the Turkish version of the Digital Competence Questionnaire (DCQ) for Nurses. The DCQ, originally developed to assess knowledge, skills, and attitudes related to digital competence in nursing, provides a comprehensive framework for evaluating how well nurses are equipped to use digital technologies in their practice [11, 25]. By adapting and validating this tool in the Turkish context, the study not only contributes to the global body of literature but also offers a practical resource for evaluating and improving digital competencies among Turkish nurses. Hence, in line with this main purpose, the following objectives have been determined;

1. To analyze the validity and reliability of the Turkish version of the DCQ for nurses.
2. To examine the exploratory and confirmatory factor structure of the Turkish version of the DCQ for nurses.

## Method

### Research design

This cross-sectional validation study aims to adapt DCQ to Turkish context, followed by its validity and reliability tests. The results of the validation phase was consistent with the guidelines of “Strengthening the Reporting of Observational studies in Epidemiology” (STROBE) checklist [26].

### Participants and settings

The current study was conducted at two public hospitals in a metropolitan city located in Turkiye’s Black Sea region using a convenience sampling method [27]. Due to time and budget constraints, as well as the challenges of accessing nurses across multiple regions of Turkiye, the study was limited to two public hospitals within a single region. The study sample consisted of nurses working in two hospitals. In Turkiye, nurses working in public hospitals are recruited through the Public Personnel Selection Examination and graduate from various universities across the country, reflecting diverse cultural and educational backgrounds. The inclusion criteria for participation were as follows: (1) nurses currently registered; (2) an age of 18 years or older; (3) being capable of using internet tools such as mobile phones or computers. Non-nurse professionals who completed the questionnaire were excluded from the analysis.

The data were collected online. Before being asked to sign an electronic informed consent, the invited registered nurses were informed about the purpose of the study, data management guidelines, anonymity, ethical principles, and publication guidelines. All participant responses were anonymized to ensure confidentiality during data collection and analysis. No personally identifiable information was recorded. All responses collected online were stored in a secure, password-protected, cloud-based system accessible only to the research team. In addition, participants were informed that their responses would only be used for research purposes and that their anonymity would be strictly maintained. Access to the dataset was restricted to the research team to prevent unauthorized access. Upon completion of the study, raw data were retained for research purposes only, following ethical and publication guidelines. The research team was actively involved in the data collection process, regularly visiting the hospitals to stay in touch with nurse managers who shared the survey link with nurses in their hospitals. This step was deemed essential to maintain integrity in data collection process and remain consistent between the two settings.

On March and April of 2024, an initial survey was administered to nurses at a public hospital in a metropolitan city located in Turkiye’s Black Sea region. The total number of nurses surveyed was 208. The survey questionnaire was presented, and data were collected through a link generated by Google Forms. The research team shared the survey link with manager nurses, who then forwarded them to unit WhatsApp groups. Participants clicked on the link to take part in the survey. Choosing to answer the questionnaire implied their consent to participate. The survey was conducted online, and all questions were set as mandatory, minimizing the occurrence of missing data. Since the profession was an open-ended question, participants who provided exclusion criteria were also excluded. Finally, 3 participants (1.44%) were excluded, resulting in a final analysis with data from 205 nurses in Sample 1 (for exploratory study). The effective response rate was 88.2%. In sample 1, the participants ranged in age from 23 to 62 (M = 43.09, SD = 8.13), and 178 of them (86.82%) were female.

On May to August 2024, a retest was conducted using the same procedures and methods as the initial survey, involving 215 participants. This phase proposed to validate the findings from Sample 1. The second survey was conducted among nurses from a public training and research hospital in the Black Sea Region Turkiye. A total of 215 data sets were collected in sample 2 (for validation study). A total of 7 questionnaires (3.26%) were excluded participants who provided exclusion criteria. The specific exclusion criteria were consistent with the initial test. Ultimately, 208 questionnaires were included for analysis, with an effective response rate of 96.74%. In sample 2, the participants ranged in age from 19 to 52 (M = 34.46 SD = 8.50), and 162 of them (77.9%) were female.

### Measurement tools

The survey included questions on demographics and individual characteristics (age, gender, highest level of completed education, marital status, units, and profession etc.) and items related to digital competence, affinity for technology interaction and attitude for digital technology. All question was mandatory and designed to exclude participants from the analysis who did not belong to the inclusion criteria.

#### Digital competence questionnaire (DCQ)

The DCQ, developed by Golz, Hahn and Zwakhalen [23] was employed for assessment. In the Delphi study, the construct validity and internal consistency of scale was not tested psychometrically [23]. This scale comprises 26 items, rated on a 5-point Likert scale, where 1 indicates “strongly disagree,” and 5 indicates “strongly agree.” The DCQ consists of 26 positive items. There are no reverse-scored items in the scale. Higher scores on the questionnaire indicate a stronger inclination toward digital competence. The S-CVI/Ave for the list of the final 26 items (knowledge, 4; skills, 8; attitude, 14) was 0.95 (SD, 0.07) for the original study.

#### Affinity for technology interaction scale (ATIS)

The ATIS was developed by Franke et al. [28] and adapted into Turkish by Demir et al. (2023). The ATIS assesses affinity for technology with a focus on user-system interaction. The ATIS consists of 9 items and is a self-report questionnaire. 3 items are reverse coded. Each item is rated on a Likert six-point scale ranging from 1 to 6, with a total score range from 9 to 54. A higher total score indicates a higher level of affinity for technology. The internal consistency coefficient in this study was 0.795 [29].

#### Attitude for digital technology scale (ADTS)

The ADTS was developed by Cabi 2016. The ADTS assesses attitude for digital technology [30]. The ADTS consists of 39 items, *eight sub-factors* and is a self-report questionnaire. In the present study, the ‘competence’ factor of the scale consisting of 10 items was used. Each item is rated on a Likert five-point scale ranging from 1 to 5, with a total score range from 9 to 54 for ADTS-competence factor. The internal consistency coefficient in this study was 0.933.

### The translation process of the DCQ

Initially, a written authorization was obtained from the developer of the scale, Dr. Golz, for using the DCQ. The method proposed by Brislin et al. was used to translate the DCQ into Turkish [31], and we employed the forward-backward translation technique for translating the scale from English into Turkish.

In the initial translation phase, we invited two experts to translate the English version of the questionnaire into Turkish. These experts hold doctoral degrees in English and have studied in English-speaking countries. They are bilingual professionals who speak both languages natively and are well-acquainted with the cultural context in which the questionnaire was administered. Furthermore, they are familiar with psychometric concepts and have participated in multiple scale translation projects. Both experts were asked to independently translate the English version of the questionnaire into Turkish while striving to maintain the original meaning as closely as possible. Subsequently, we compared and merged their translations. Any discrepancies were discussed and resolved by both experts, resulting in the preliminary Turkish version of the questionnaire.

In the back-translation phase, we invited an expert who speaks both Turkish and English at a native level and holds a doctorate in nursing to translate the preliminary Turkish version back into English. This expert had no prior involvement in the initial translation process and was unfamiliar with the original English questionnaire, ensuring an unbiased back-translation. Their linguistic proficiency and academic background contributed to an accurate and contextually appropriate translation, facilitating a rigorous comparison with the original English version. Subsequently, the researchers compared the back-translated English version with the original English version to identify any discrepancies. To further ensure linguistic and conceptual equivalence, a native English speaker was invited to evaluate both English versions and confirm the absence of significant differences. Following this process, the finalized Turkish version of the questionnaire was reviewed by two experts in psychometrics to assess its accuracy and applicability. Special attention was given to cultural differences and their potential impact on the scale’s validity within the Turkish context. Finally, the back-translated version was sent to the corresponding author of the original scale, and an opinion was obtained.

### Pre-test

To further enhance the comprehensibility, clarity, and cultural appropriateness of the Turkish adaptation of the DCQ, cognitive interviews were conducted with a sample of 30 nurses. This process aimed to systematically assess whether the translated items were accurately interpreted in alignment with the original version, thereby ensuring conceptual equivalence, linguistic precision, and contextual relevance [32]. The study employed a 5-point Likert-type scale for item responses, ranging from (1) “strongly disagree” to (5) “strongly agree.” To mitigate potential biases, the items were presented in randomized order. During the pre-test, participants were asked to articulate their understanding of each item, allowing the researchers to assess the clarity and interpretability of the statements in line with established methodologies [32]. Based on nurses’ feedback, the researchers made necessary adjustments to improve the wording and structure of the questionnaire items. Following these revisions, the finalized version of the instrument was prepared for use in the main study, ensuring its suitability for exploratory factor analysis (EFA). The Turkish version of the DCQ is presented as Supplementary Material.

### Statistical analysis

We performed statistical analyses using R (Version 4.4.1) and RStudio (Version 2023.12.1 + 402) (Posit Software, Boston, MA, USA). Construct validity was assessed using “lavaan” [33] and “semTools” [34] in R. Exploratory Factor Analysis (EFA), Convergent validity, items analysis, and reliabilities were assessed using JASP software (Version 0.18.1) [35]. We conducted an exploratory factor analysis (EFA) for the factor structure of the Digital Competence Questionnaire. Factor analysis reveals the intrinsic structure among variables [36, 37]. The assumptions for an EFA were item correlations above 0.60, Kaiser–Meyer–Olkin values ≥ 0.70 and a significant Bartlett’s test of sphericity for the included items and all items combined [37].

Confirmatory factor analysis (CFA) was used to test whether the current data fits the structural model from the EFA and the original scale. CFA assesses the reliability of the theoretical model by comparing the fit between the observed data and the theoretical model [38]. The multivariate normality was measured using Mardia’s multivariate kurtosis [39]. Multivariate normality was tested with Mardia’s MVN test, and both Mardia’s multivariate skewness test (skewness = 92.920, χ²_(969)_ = 3221.230, *p* < 0.001) and Mardia’s multivariate kurtosis test (kurtosis = 478.996, Z = 44.259, *p* < 0.001) showed that the data did not have a multivariate normal distribution.

Given the ordinal nature of the data, all items measured on a 5-point Likert scale were analyzed as categorical indicators within the theoretical model. Accordingly, the diagonally weighted least squares (DWLS) method was employed for parameter estimation. Model fit was assessed using the Root Mean Square Error of Approximation (RMSEA), with acceptable values ranging between 0 and < 0.06; the Comparative Fit Index (CFI) and Tucker-Lewis Index (TLI), with values ≥ 0.95 indicating a good fit; and the Standardized Root Mean Square Residual (SRMR), with acceptable values ranging between 0 and < 0.08 [40]. Initially, reliability testing and item analysis were performed on the sample 2. Item-total correlations and reliability assessments were utilized for item selection. The structure of the original 26-item the Digital Competence Questionnaire was validated through both exploratory and confirmatory factor analyses. Convergent validity was established using the Affinity for Technology Interaction Scale and the Attitude for Digital Technology Scale. Reliability was further evaluated using Cronbach’s alpha (α), McDonald’s omega (ω), and test-retest reliability measures to ensure the robustness of the instrument.

## Results

Table 1 presents the demographic information of the participants in sample 1(for exploratory study) and sample 2 (for validation study). The majority of participants were female, accounting for 86.83% for sample (1) The majority of participants were female, accounting for 77.9% for sample (2) The majority of participants were in Critical Care Units in each sample. The vast majority of participants reported a bachelor’s degree for the highest level of completed education.

**Table 1:** Demographic characteristics of the participants

Baseline characteristic	Exploratory (*N* = 205)	Validation (*N* = 208)
	*n* (%) / M(SD)	*n* (%) / M(SD)
Age (years)*	43.09 (*8.13*)	34.46 (*8.50*)
Professional (years)*	21.75 (*9.42*)	11.80 (*9.62*)
Institutional work (years)*	11.57 (*9.09*)	6.14 (*6.39*)
**Gender**		
Female	178 (86.83)	162 (77.9)
Male	27 (23.17)	46 (22.1)
**Marital status**		
Married	170 (82.93)	112(53.8)
Single	35 (17.07)	96 (46.2)
**Highest level of completed education**		
High school	12 (5.85)	11 (5.3)
Pre-Bachelors	26 (12.68)	26 (12.5)
Bachelors	145 (70.73)	142 (68.3)
Postgraduate and above	22 (10.73)	29 (13.9)
**Units**		
Critical Care Units	58 (28.29)	69 (33.2)
Inpatient Clinics	61 (29.76)	69 (32.2)
Outpatient Clinics	40 (19.51)	21 (10.1)
Administration	46 (22.44	49 (23.6)
**Digital competency training**		
Yes	107 (52.20)	83 (39.9)
No	98 (47.80	125 (60.1)
**In-service training sufficient**		
Sufficient	65 (60.75)	44 (53.0)
Insufficient	42 (39.25)	39 (47.0)

*: Mean and standard deviation

M: Mean; SD: standard deviation

### Research items results

Table 2 summarizes the descriptive results of the 26 items. The median of the items ranged between 2 and 4, with high scores indicating a ceiling effect. Skew and kurtosis were not found to be above the cut-offs with < ± 2 for skewness (-1.70- 0.30) and < ± 7 for kurtosis (-0.80–4.69).

**Table 2:** Descriptions of the items

Items	Topic	Median	Mean	SD	Min	Max	Skew	Kurtosis
Q1	Knowledge	4	3.53	0.94	1	5	-0.77	0.28
Q2	Knowledge	4	3.80	0.84	1	5	-1.21	2.08
Q3	Knowledge	4	4.08	0.88	1	5	-1.58	3.31
Q4	Knowledge	2	2.66	1.08	1	5	0.30	-0.80
Q5	Skills	4	3.73	0.85	1	5	-0.65	0.15
Q6	Skills	4	3.88	0.73	1	5	-1.01	1.82
Q7	Skills	4	3.94	0.75	1	5	-1.17	2.61
Q8	Skills	4	3.96	0.70	1	5	-1.43	4.05
Q9	Skills	4	4.08	0.67	2	5	-0.89	2.09
Q10	Skills	4	4.01	0.66	2	5	-0.85	2.00
Q11	Skills	4	4.02	0.65	2	5	-0.76	1.77
Q12	Skills	4	4.01	0.64	2	5	-0.57	1.28
Q13	Attitude	4	4.35	0.67	1	5	-1.35	4.69
Q14	Attitude	4	3.69	1.03	1	5	-0.98	0.55
Q15	Attitude	4	3.94	0.79	1	5	-0.72	0.79
Q16	Attitude	4	3.95	0.76	1	5	-0.81	1.30
Q17	Attitude	4	3.92	0.84	1	5	-1.04	1.58
Q18	Attitude	4	4.32	0.81	1	5	-1.70	4.27
Q19	Attitude	4	4.22	0.64	2	5	-0.68	1.46
Q20	Attitude	4	4.24	0.63	2	5	-0.47	0.50
Q21	Attitude	4	4.02	0.73	2	5	-0.49	0.25
Q22	Attitude	4	4.20	0.62	2	5	-0.53	1.20
Q23	Attitude	4	4.30	0.57	2	5	-0.27	0.33
Q24	Attitude	4	4.11	0.69	1	5	-1.16	3.93
Q25	Attitude	4	4.19	0.71	1	5	-0.96	2.22
M26	Attitude	4	4.28	0.66	2	5	-0.69	0.81

### Structural validity

EFA was used to explore the factor structure of the DCQ. For EFA, the Principal Axis Factor extraction method and scree plot and parallel analysis were used to determine the number of factors [37, 41]. Since all items in the scale are related to digital competence, it was thought that there would be a correlate between the factors that would emerge after the factor analysis, and therefore “oblimin” rotation, which is one of the oblique rotation methods, was selected [37, 42].

Kaiser’s measure of sampling adequacy for EFA was found to be 0.89, and Bartlett’s test of sphericity yielded significant results, χ²_(136)_ = 2553.746, *p* < 0.001. These findings indicate that the dataset is suitable for EFA [42]. In the factor extraction process, eigenvalues were examined first. Additionally, the scree plot [43] was employed as part of the analysis. Based on these procedures, the scale was identified to have a two-factor structure.

EFA indicated that five items either demonstrated cross-loadings on multiple factors with a difference of less than 0.10 or failed to load significantly on any single factor (loading < 0.60) [44]. The two-factor structure explains 59.8% cumulative variance, which resulted from the EFA phase after omitting such nine items. The two-factor structure named Knowledge-Skills, and Attitude constituted 28.5% and 31.3% of the explained variance, respectively. All commonalities were higher than 0.26. The items in the first factor consisting of 8 items are between 0.657 and 0.811. The factor loads of the items in the second factor consisting of 9 items are between 0.602 and 0.849 (Table 3). Also, Conbach’s alpha internal consistency coefficients of the factors were also reviewed. The rotated factor matrix is presented in Table 3.

**Table 3:** Rotated factor matrix

Items	Knowledge and Skills	Attitude
Q9	0.811	
Q11	0.810	
Q12	0.806	
Q10	0.775	
Q7	0.772	
Q6	0.746	
Q8	0.669	
Q2	0.657	
Q23		0.849
Q22		0.831
Q20		0.810
Q19		0.795
Q21		0.775
Q26		0.770
Q25		0.722
Q24		0.611
Q15		0.602
Eigenvalue	2.561	8.399
Total variance explained (%59.8)	0.285	0.313
Cronbach’s α/ McDonald’s ω	0.910/0.905	0.928/0.928
Kaiser-Meyer-Olkin Measure of Sampling Adequacy 0.916		
Bartlett's Test of Sphericity Sig. <0.01		

**Fig. 1:**
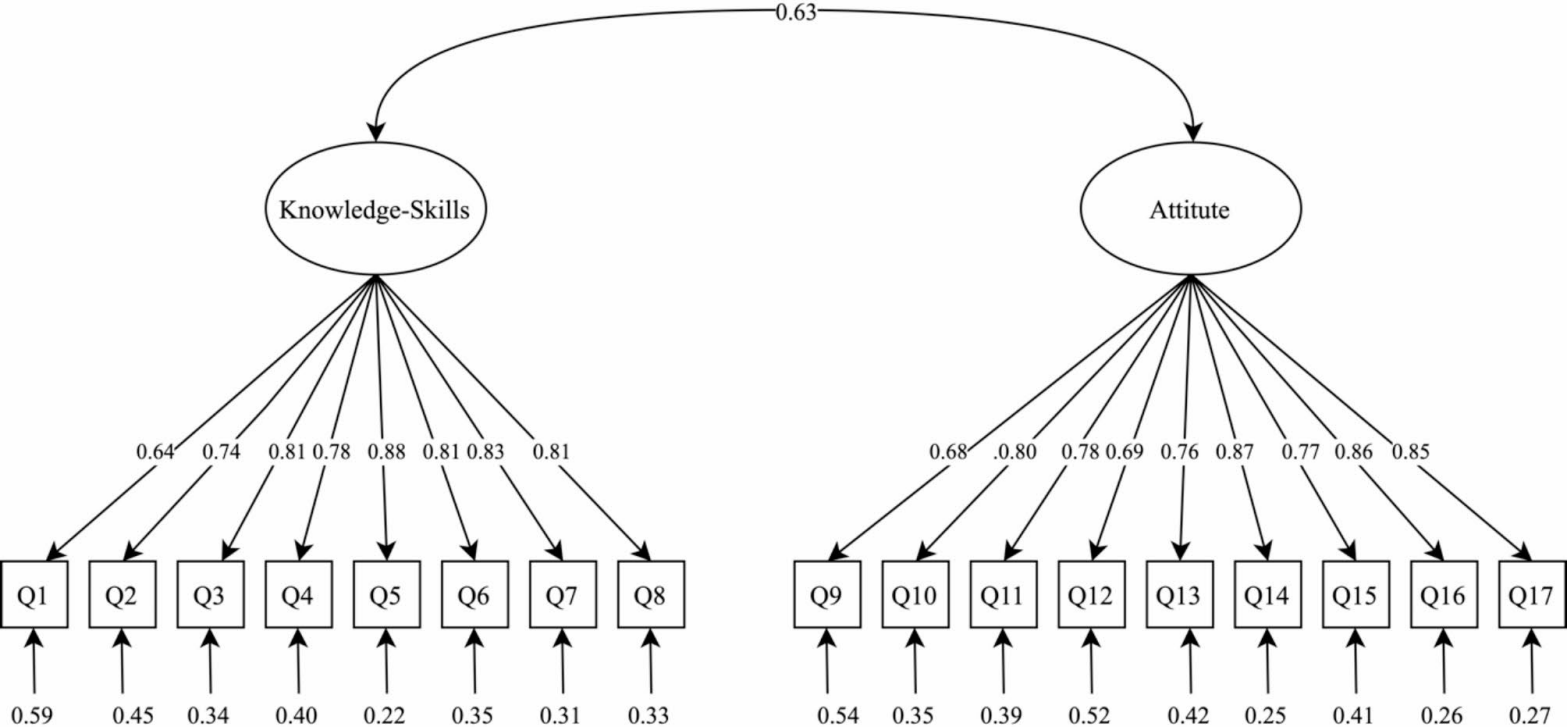
Measurement model path diagram of the DCQ

CFA was conducted to assess the model fit of the Turkish version of the 17–item DCQ. Item analyses were performed before CFA. There is no specific standard for the item total correlation coefficient. Although it is stated that item-total correlation coefficient values of 0.50 and/or above are significant, in practice, correlations are mostly expected to be non-negative and above 0.20 [45]. The corrected item-total correlation ranged from 0.552 to 0.761, indicating correlation coefficient values of 0.50 above. Factor loadings ranged from 0.637 to 0.882, indicating a well-structured scale. The items were fit into a two-factor, with the model fit indices yielding χ²/df = 1.47, RMSEA = 0.030 (90% CI 0.020–0.039), SRMR = 0.059, CFI = 0.992, and TLI = 0.991. All these indices met acceptable model fit criteria. Factor loadings, descriptive statistics, and item-total score correlations are presented in Table 4. The path diagram of the measurement model for the DCQ is presented in Fig. 1.

**Table 4:** Descriptive statistics for the DCQ items* (*n* = 208)

Items	Factor loadings	Mean	SD	Item-total correlations
1. I am familiar with digital…	0.637	3.577	0.898	0.552
2. I feel confident about…	0.743	3.803	0.821	0.633
3. I feel confident about using…	0.810	3.808	0.801	0.694
4. I am able to reach conclusions…	0.776	3.894	0.750	0.666
5. I feel confident about using…	0.882	3.788	0.748	0.755
6. I feel confident secure…	0.807	3.837	0.725	0.691
7. I feel confident…	0.831	3.827	0.763	0.705
8. I feel confident about…	0.809	3.745	0.773	0.693
9. Digital technology fits…	0.682	3.817	0.865	0.620
10. I believe….quality of care.	0.803	3.981	0.786	0.714
11. I believe….clinical care.	0.783	3.889	0.794	0.697
12. I believe that…outcomes.	0.690	3.793	0.774	0.618
13. I believe…. patients.	0.760	3.909	0.746	0.687
14. believe that…. professionals.	0.865	4.077	0.705	0.761
15. I like to use digital…	0.770	3.957	0.806	0.688
16. I am keen to use new digital…	0.860	4.067	0.777	0.755
17. I believe that digital…	0.852	4.034	0.789	0.752

Note: All factor loadings *P* < 0.001; * DCQ items translated to English

### Convergent validity and reliability

CR and AVE were used to assess the convergent validity of the scale [46]. AVE values greater than 0.50 indicate adequate convergence [47]. The DCQ, CR values greater than 0.70, and AVE values greater than 0.50 indicate acceptable convergence.

The total score of the Turkish version of the 17–item DCQ was positive and moderately correlated with the ATIS and ADTS scores (see Table 5). The Turkish version of the 17-item DCQ demonstrated high internal consistency reliability. Cronbach’s α was 0.944 for the overall scale (95% CI [0.931–0.954], n = 17 items), 0.912 for the “Knowledge & Skills” subscale (95% CI [0.910–0.941], n = 8 items), and 0.937 for the “Attitude” subscale (95% CI [0.923–0.949], n = 9 items). McDonald’s ω = 0.942 for the overall scale and between 0.926 and 0.937 for the sub-factor; CR = 0.965 for the overall scale. CR is a less biased reliability estimate than Cronbach’s alpha, and CR values of 0.70 and above are considered acceptable [47, 48]. Correlation analysis results between DCQ sub-factor’s total scores and calibration standards are presented in Table 5.

**Table 5:** Correlation analysis results between DCQ sub-factors total scores and calibration standards

	M(SD)	Cronbach’s α (95% CI)	CR	AVE	1	2	3
DCQ (full scale)	65.80 (9.68)	0.944 (0.931–0.954)	0.965				
^1^DCQ- Knowledge and Skills	30.28 (5.11)	0.912 (0.910–0.941)	0.929	0.624	**(0.790)**		
^2^DCQ-Attitude	35.52 (5.75)	0.937 (0.923–0.949)	0.936	0.620	0.587**	**(0.788)**	
^3^ATIS					0.548**	0.608**	-
^4^ADTS-Competence					0.696**	0.606**	0.604**

Notes: Bold values in parentheses indicate the square root of AVE.

**P < 0.01

### Test-retest reliability

**Fig. 2:**
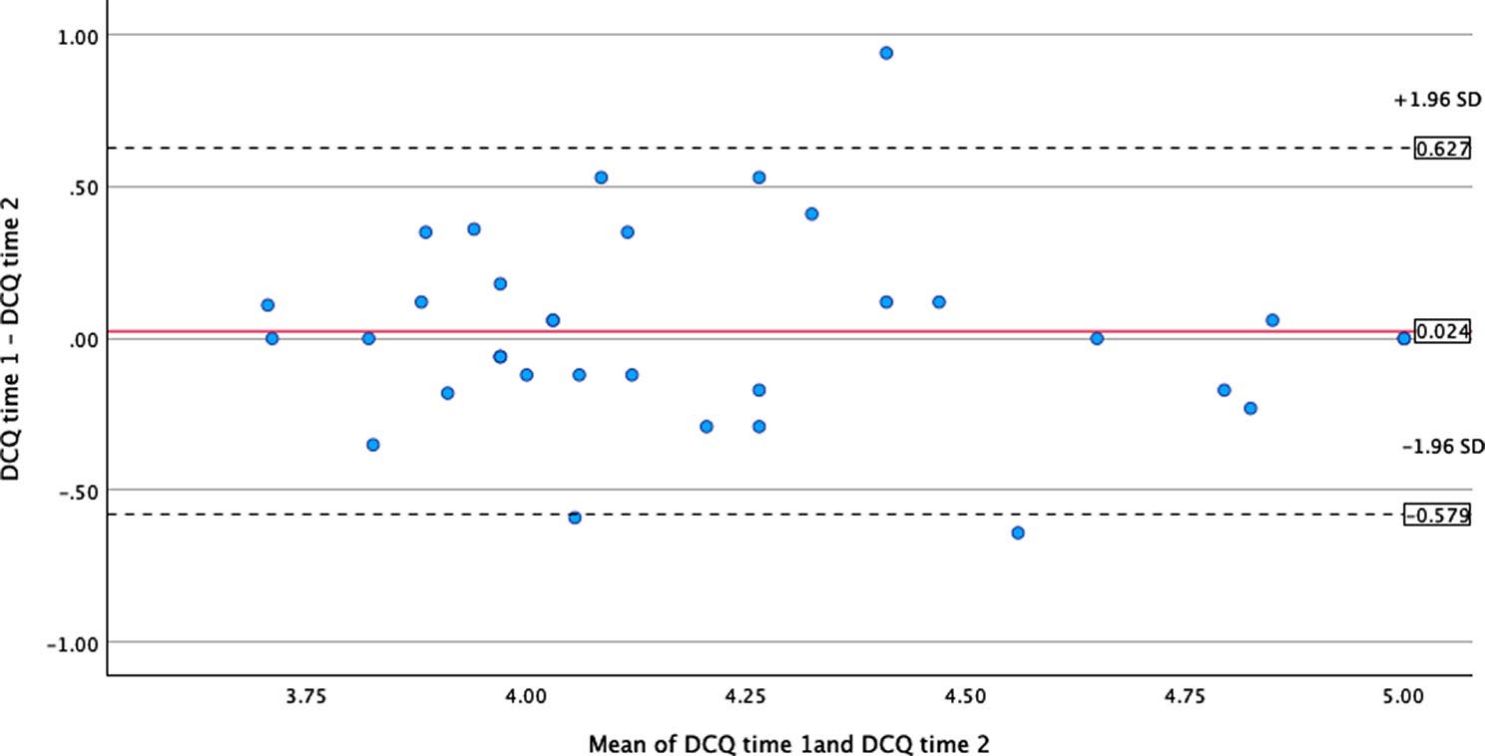
The Bland-Altman test for test- retest reliability of DCQ

The Turkish version of the 17-item DCQ test-retest reliability was good, with scale-level intra-class correlation ranging 0.735–0.887 and average inter-item correlation ranging 0.502–0.629. Test-retest reliability estimates for the full DCQ and each sub-factor are presented in Table 6. Finally the analysis of test-retest reliability among 36 nurses using the Bland-Altman test for agreement is shown in Fig. 2. Bland–Altman analyses showed a mean difference between test and retest total scores of 0.024 (SD = 0.308). The 95% confidence interval (CI) for the mean difference was − 0.080 to 0.128. The upper and lower limits of agreement (LoA) for total scores were 0.627 (95% CI [0.446, 0.807]) and − 0579 (95% CI [-0.760, -0.399]), respectively.

**Table 6:** Descriptive statistics and reliability estimates for the full DCQ and each sub-factor

	Mean scores (SD)^a^	AIC^b^	ICC (95% CI)^c^
Knowledge and Skills	4.18 (0.43)	0.621	0.887 (0.783.–0.944.)
Attitude	4.28 (0.43)	0.629	0.735 (0.495–0.869 )
DCQ (full scale)	4.23 (0.38)	0.502	0.837 (0.686–0.920 )

Notes

^a^Mean scores are transformed to a range from 0–5

^b^*n* = 208; AIC = Average inter-item correlation

^c^*n* = 36; ICC = Intra-class correlation

## Discussion

The exponential growth of digital technologies has transformed industries worldwide, with healthcare experiencing one of the most profound impacts. The ongoing digitalization of the nursing sector offers numerous benefits, including improved workflows and more efficient communication. However, nurses need digital competencies to use new technologies effectively. Nursing staff members who will particularly benefit from digitalization include those involved in patient process organization, treatment plan improvement, and access to health information. Additionally, digitalization improves patient care by enabling the delivery of sensitive and individualized care while reducing the workload of nursing staff [49]. Therefore, it is crucial that nurses possess the requisite competencies and expertise in the utilization of digital technologies to facilitate the integration of the roles, relationships, responsibilities, and ethical and professional dilemmas of all professionals involved in the healthcare field.

There is a general consensus within the healthcare sector that professionals in various fields require a certain level of digital competence, or digital literacy. Nevertheless, the precise scope and metrics for assessing the digital competence of healthcare professionals remain undetermined [50]. Digital competence is the capacity to consistently combine and utilize relevant knowledge, skills, and psychosocial elements (such as beliefs, attitudes, values, and motivations) to perform effectively within a specific domain [51] and the necessity of digital competence in healthcare is becoming increasingly apparent. As digital competence has been identified as a factor that mitigates the experience of technology-related stress at work [52], it is a key consideration in the development of effective stress management strategies. Therefore, imperative that research be conducted on nurses’ digital competence.

Digital competence can be measured using self-reported questionnaires. Several tools are available to measure digital competence in different populations [18–22]. Some of these tools measured nurses’ digital competence [25, 53]. A positive attitude towards technology at work in healthcare is associated with successful adoption and use at work. The shortcomings of existing questionnaires in measuring nurses’ digital competence include focusing only on knowledge and skills and neglecting attitudes. The Digital Competence Questionnaire developed by Golz et al. (2023) overcomes this shortcoming in measuring knowledge, skills, and attitudes.

The present study involved the adaptation of the DCQ developed by Golz et al. (2023) into a Turkish version providing an effective measurement tool tailored to Turkish nurses’ digital competence, thereby facilitating research on digital competence in Turkiye. This is particularly important because there was no previously existing digital competency assessment tool expressly designed for nurses in Turkiye, that was both valid and trustworthy. Golz et al. (2024) examined the psychometric properties of the DCQ and exploratory analyses of the scale, but did not perform a CFA. The EFA results revealed the two-factor structure of the Turkish version with attitude factor (*n* = 9 items) and knowledge and skills factor (*n* = 8 items) consistent with the two-factors structure of the original scale [24]. CFA results in different sample groups confirmed the two-factor structure of the DCQ. Factor attitude assesses the participants’ attitude and factor knowledge and skills, captures the knowledge and skills. Based on the data collected, it was impossible to distinguish between knowledge and skills, although theoretically they are two different entities of digital literacy [54]. Both factors fit the underlying theory of digital competence. The first factor, “attitude,” assesses nurses’ attitudes toward digital competence in clinical settings, while the second factor, “knowledge and skills,” evaluates their digital knowledge and skills.

To assess the convergent validity of the DCQ, the affinity for technology interaction and the level of attitude toward digital technology among nurses from the same study group were also evaluated. Therefore, a relationship between digital competence, affinity for technology interaction and attitude toward digital technology is expected. Previous studies show that there is a relationship between attitudes towards using digital technologies and digital competence [55, 56]. Correlation analysis reveals a significant correlation between digital competence scores and affinity for technology interaction (technology skills), attitude toward digital technology. This finding shows that the scale has achieved convergent validity.

The results of this study demonstrate that the Turkish version of the Digital Competence Questionnaire (DCQ) exhibits high reliability, as evidenced by internal consistency and test-retest reliability analyses. Internal consistency, a key indicator of scale reliability, ensures that the items within a measure are cohesively aligned to assess the intended construct [57]. Similarly, test-retest reliability provides critical evidence of a scale’s temporal stability, reflecting its capacity to produce consistent results over time [58].

To test the reliability of the DCQ, internal consistency was first examined. For this reason, Cronbach’s α coefficient and item-total correlations were analyzed. In this study, the Cronbach’s α coefficient of the scale for the factor “knowledge & skills” with 0.912 and for the factor “attitude” with 0.937 was found to be high. Consistent with previous research, the internal consistency results indicate that the scale effectively captures the underlying constructs of “knowledge and skills” and “attitudes” [24]. Also, in the current study, the item-total correlations were between 0.552 and 0.761. These findings indicate that the scale has internal consistency.

In this study, test-retest reliability was assessed by administering the DCQ to a sample of 36 nurses with a one-month interval. The results demonstrated strong agreement between the two administrations, indicating that the scale maintains its reliability over time [59]. Sub-factor reliability analysis further confirmed that the scale effectively captures different dimensions of digital competence with stable and consistent measurements. These findings demonstrate that the ICC values consistently reflect robust test-retest reliability, underscoring the scale’s stability and its ability to produce consistent results across repeated measurements over time.

Furthermore, the Bland–Altman analysis provided additional support for the stability of the Turkish DCQ by examining the agreement between the first and second administrations. The minimal variability observed across repeated measurements highlights the scale’s ability to yield consistent results over time, reinforcing its applicability for longitudinal assessments of digital competence. The high degree of agreement between the two administrations indicates that the scale can reliably measure digital competence across different time points [60]. The results also emphasize the suitability of the DCQ to assess nurses’ digital competencies in clinical practice in the Turkish context. Digital competence is increasingly recognized as an essential skill for healthcare professionals, especially in the era of digital health transformations [61]. Previous studies emphasize that reliable and valid tools are critical to identify competency gaps and design targeted interventions to improve digital literacy [50]. The results of this study confirm that the Turkish DCQ meets these requirements, making it an important resource for future research and practice.

### Limitations

While this study makes valuable contributions by adapting and validating the DCQ self-report scale for the Turkish context, several limitations should be acknowledged to ensure a comprehensive understanding of the findings and their generalizability (external validity). Firstly, the representativeness of the sample is a notable limitation. The convenience sampling may have resulted in a relatively homogeneous sample, potentially influencing the reliability and generalizability of the findings. The absence of a random sampling strategy introduces the possibility of selection bias, which may have impacted the diversity of the sample frame. To provide further context on the sample’s composition, most participants were predominantly female, which aligns with Turkiye’s demographic distribution of the nursing workforce. However, the lack of randomization remains a limitation that should be considered when interpreting the findings. Future studies should employ probability sampling methods and include a more diverse participant pool, particularly with a greater representation of male nurses and those from different professional backgrounds and geographical regions, to enhance the generalizability of the results. Secondly, despite employing an anonymous survey approach and standardized scales while emphasizing the absence of correct or incorrect responses, the self-report nature of the study remains subject to potential response biases. Participants may have provided socially desirable answers or may have misjudged their own digital competencies, which could affect the accuracy of the results. Finally, the study was conducted within the specific cultural context of Turkiye, which may shape nurses’ perceptions of digital technologies and their self-efficacy in utilizing them. Cultural norms, values, and systemic factors specific to Turkiye could influence these perceptions, potentially limiting the applicability of the findings to other professional groups or cultural contexts. Future research should explore whether the structure and validity of the scale hold across diverse professional and cultural groups to ensure broader applicability.

## Conclusion

This study utilized a rigorous methodological approach to validate the Turkish version of the DCQ among registered nurses. The Turkish version of the DCQ demonstrated strong reliability and validity, with significant associations observed between digital competence and constructs such as affinity for technology interaction and attitudes toward digital technology. The validated DCQ provides a robust foundation for future research into the digital competencies of nurses. As a 17-item self-report scale, the DCQ offers a practical and specific tool for assessing digital technology-related competencies among Turkish nurses. It addresses the growing need for targeted evaluations of nurses’ capacities to effectively manage digital technology-related tasks within their professional roles. Furthermore, the DCQ has the potential to support the assessment of digital competence in clinical practice and contribute to raising digital awareness within healthcare teams. Future research should aim to validate the psychometric properties of the DCQ across different national contexts and professional groups. Such efforts will enhance the generalizability of the instrument and provide valuable insights into the digital competence of healthcare professionals worldwide.

## Electronic supplementary material

Below is the link to the electronic supplementary material.

Supplementary Material 1

## Data Availability

The datasets generated and analysed during the current study are not publicly available due but are available from the corresponding author on reasonable request.
