# Supplementary material for: Reliability and validity of the Turkish version of the digital competence questionnaire for nurses
Source: BMC Nurs. 2025 Sep 30;24:1225. doi: 10.1186/s12912-025-03052-6 (PMC12486577; doi:10.1186/s12912-025-03052-6)
Supplement: Supplementary file 1 — Supplementary Material 1 [file 12912_2025_3052_MOESM1_ESM.docx]

**Supplementary Material –The Digital Competence Questionnaire (DCQ)**

| **Items** | **Strongly disagree** | **Disagree** | **Undecided** | **Agree** | **Strongly agree** |
| --- | --- | --- | --- | --- | --- |
| I am familiar with digital technologies at my workplace |  |  |  |  |  |
| I feel confident about using digital technology to share relevant information. |  |  |  |  |  |
| I feel confident about using digital technology to obtain data and information on clinical care. |  |  |  |  |  |
| I am able to reach conclusions based on information acquired through digital technologies. |  |  |  |  |  |
| I feel confident about using digital technology to communicate. |  |  |  |  |  |
| I feel confident about the secure management of health data using digital technology. |  |  |  |  |  |
| I feel confident about using digital technology to find relevant information. |  |  |  |  |  |
| I feel confident about using digital technology. |  |  |  |  |  |
| Digital technology fits well with the way I like to work. |  |  |  |  |  |
| I believe that digital technology provides numerous benefits in terms of quality of care. |  |  |  |  |  |
| I believe that digital technology improves clinical care. |  |  |  |  |  |
| I believe that digital technology improves patient outcomes. |  |  |  |  |  |
| I believe that digital technology is beneficial for my patients. |  |  |  |  |  |
| I believe that digital technology is beneficial for healthcare professionals. |  |  |  |  |  |
| I like to use digital technology at work. |  |  |  |  |  |
| I am keen to use new digital technologies in my future professional practice. |  |  |  |  |  |
| I believe that digital technology is relevant for my future profession. |  |  |  |  |  |
